# Predicting ICU Mortality Among Septic Patients Using Machine Learning Technique

**DOI:** 10.3390/jcm14103495

**Published:** 2025-05-16

**Authors:** Aisha A. Al-Ansari, Fatima A. Bahman Nejad, Roudha J. Al-Nasr, Johayra Prithula, Tawsifur Rahman, Anwarul Hasan, Muhammad E. H. Chowdhury, Mohammed Fasihul Alam

**Affiliations:** 1 Department of Public Health, College of Health Sciences, QU Health, Qatar University, Doha 2713, Qatar; aa1603886@qu.edu.qa (A.A.A.-A.); fb1517563@qu.edu.qa (F.A.B.N.); ra1602873@qu.edu.qa (R.J.A.-N.); 2 Department of Electrical and Electronics Engineering, University of Dhaka, Dhaka 1000, Bangladesh; prithulajohayra@gmail.com; 3 Department of Biomedical Engineering, Johns Hopkins University, Baltimore, MD 21218, USA; tawsifurrahman.1426@gmail.com; 4 Department of Mechanical and Industrial Engineering, College of Engineering, Qatar University, Doha 2713, Qatar; ahasan@qu.edu.qa; 5 Department of Electrical Engineering, College of Engineering, Qatar University, Doha 2713, Qatar

**Keywords:** sepsis, ICU mortality, prediction, machine learning, over-sampling, augmentation

## Abstract

**Introduction**: Sepsis leads to substantial global health burdens in terms of morbidity and mortality and is associated with numerous risk factors. It is crucial to identify sepsis at an early stage in order to limit its escalation and sequelae associated with the condition. The purpose of this research is to predict ICU mortality early and evaluate the predictive accuracy of machine learning algorithms for ICU mortality among septic patients. **Methods**: The study used a retrospective cohort from computerized ICU records accumulated from 280 hospitals between 2014 and 2015. Initially the sample size was 23.47K. Several machine learning models were trained, validated, and tested using five-fold cross-validation, and three sampling strategies (Under-Sampling, Over-Sampling, and Combination). **Results**: The under-sampled approach combined with augmentation for the Extra Trees model produced the best performance with Accuracy, Precision, Sensitivity, Specificity, F1-Score, and AUC of 90.99%, 84.16%, 94.89%, 88.48%, 89.20%, and 91.69%, respectively, with Top 30 features. For Over-Sampling, the Top 29 combined features showed the best performance with Accuracy, Precision, Sensitivity, Specificity, F1-Score, and AUC of 82.99%, 51.38%, 71.72%, 85.41%, 59.87%, and 78.56%, respectively. For Down-Sampling, the Top 31 combined features produced Accuracy, Precision, Sensitivity, Specificity, F1-Score, and AUC of 81.78%, 49.08%, 79.76%, 82.21%, 60.76%, and 80.98%, respectively. **Conclusions**: Machine learning models can reliably predict ICU mortality when suitable clinical predictors are utilized. The study showed that the proposed Extra Trees model can predict ICU mortality with an accuracy of 90.99% accuracy using only single-entry data. Incorporating longitudinal data could further enhance model performance.

## 1. Background

Sepsis is a medical emergency characterized by the body’s systemic immune response to an infectious process, which may lead to organ dysfunction and death [1]. Data acquired for this research were in line where sepsis is defined as the presence of an infection and detection of two or more Systemic Inflammatory Response Syndrome (SIRS) criteria, which places more priority on signs and symptoms of the disease [2]. The prevalence of sepsis is anticipated to continue growing, particularly among patients with pre-comorbidities, making the issue a continuing concern [3,4]. Early prediction of sepsis is crucial to avoid sepsis mortality, as the probability of deaths significantly increases with each hour delay in administering antibiotics [5].

Intensive Care Unit (ICU) mortality among septic patients is on the rise, so it is essential to prevent patients’ conditions from deteriorating and to administer treatments expeditiously, since each hour of delayed treatment is related with a 3.6–9.9% increase in fatality [3]. Worldwide, the World Health Organization (WHO) projected 48.9 million sepsis cases and 11 million sepsis-related mortalities, which account for 20% of all death tolls [5]. The number of patients with severe sepsis increased from 27% to 41%, but the death rate decreased by just 2.7%, remaining at 24.1% [6]. Additionally, sepsis became the leading cause of ICU admission and mortality [7]. Multiple studies have indicated that men had a 70% greater risk of sepsis-related mortality than females [8,9,10]. A variety of factors are sources of sepsis, involving community-acquired infections (over 60%), healthcare-associated infections (26%), and hospital-acquired infections (11%). In total, 64% (pneumonia), 20% (abdominal), 14% (gastro and urinary), and 14% (intestinal and urinary) are the most prevalent regions of infection among septic patients who were admitted to the ICU [11].

Due to the direct and indirect expenses of the disease, sepsis may place a substantial monetary burden on society. The direct cost of sepsis hospital charges in the United States was estimated to be USD 24 billion in 2013 [12,13]. Moreover, sepsis imposes a substantial indirect cost on patients; for instance, a decrease in productivity due to absence from work, in addition to impairment days and retirement at an early age. However, data on this aspect is rare and the financial strain on society was approximated to be between USD 484 and USD 686 million per year for 6700 to 9500 patients [14].

The natural physiological and immunological reaction of the human body is the eradication of any invading infection. Due to pathogen stimulation, normal homeostasis becomes unbalanced in sepsis [15]. If somehow the bacteria penetrates into the blood circulation, it will produce toxins that trigger inflammatory responses, such as cytokines and platelet activation factors [16]. In addition, septic patients will encounter a rapid apoptosis of lymphocytes, an upsurge in coagulation, and a decline in fibrinolytic activity, which will direct an aggregate or plaque buildup and poor circulation. As a consequence, the fluid will dissipate and the patient will become hypotensive, which signifies that some organs are not being perfused, thereby resulting in tissue hypoxia [14,16].

SIRS was the primary diagnostic criterion, and patients were categorized as septic if they fulfilled two of four criteria: hyper or hypothermia, tachycardia, tachypnea, leukocytosis, or leukopenia. SIRS might detect sepsis in its earliest stages, prior to the development of further problems or the onset of septic shock and organ failure. However, these deficits in homeostasis might be attributed to medical conditions other than sepsis; hence, the Acute Physiology and Chronic Health Evaluation (APACHE) score and Sequential Organ Failure Assessment (SOFA) Score were subsequently adopted for ICU patients [17]. SOFA provides a more comprehensive scoring method used to evaluate the functionality of 12 human organs: the neurologic, liver, blood, hemodynamic, and kidneys [18]. In addition, SOFA is acclaimed for predicting ICU mortality for septic patients admitted to the ICU, which allows for a more accurate prognosis than other grading systems [19].

For the diagnosis and management of sepsis, a criterion is employed to define the sickness stage among patients, which will dictate the treatment strategy. Managing patients with multi-organ disease like sepsis needs interpretation of multiple interlayers of information. Physicians integrate these data into clinical scores, and individual judgement is based on data, physical and established clinical guidelines [20]. However, sepsis patients are managed by clinicians in ICU wards where their work is organized in shifts, and experiences and clinical judgements might vary significantly between physicians. Therefore, more objective measures of patients and disease trajectories could lead to better health outcomes and decision processes.

While dealing with sepsis patients, a particular challenge for physicians is to make decisions when to stop treatment [20,21,22,23,24]. Following the clinical guidelines, patients who do not benefit from the ICU should not be admitted. However, making this critical judgement by a physician could be impossible at admission when only limited data are available for a patient. Additionally, during that time, physicians need to assess further information on concomitant diseases, pre-admission comorbidities and frailty, and responsiveness to treatment. In reality, often physician’s decisions on ICU services or any treatment restrictions are made after consulting with other healthcare providers, the patient, and their family [24]. Nevertheless, given a large volume of information generated from a septic patient’s ICU stay, making this judgment is often tricky, and outcome prediction remains challenging. Consequently, machine learning methods that intrinsically integrate and process a large amount of data could play an important role in facilitating clinical decision making.

A high prevalence of sepsis-related ICU death was attributed to poor blood circulation, tissue perfusion damage, and the onset of organ failure; however, the ICU mortality rate was greater among patients with failure in four or more organs than among those with failure in one organ: 60.8% against 9.2%. Research found a correlation between high ICU death rates and lung infection (34.7%). In patients with positive cultures, Gram-positive organism’s “staphylococcus” and Gram-negative species “Pseudomonas and Acinetobacter” were shown to be related with increased ICU mortality (56%, 49.5%, and 44.9%, respectively). Other independent risk factors reported to be related with sepsis include a high APACHE II score, concomitant cardiovascular illnesses, malignancies, and the use of Renal Replacement therapy [25].

A study demonstrated that ICU mortality is higher among septic patients with elevated SOFA scores (90% sensitivity and 80% specificity); additionally, it was observed that patients receiving invasive mechanical ventilation (IMV) had a seven-fold higher rate of ICU mortality than those receiving non-invasive mechanical ventilation (NIMV) due to the growth of multidrug-resistant microorganisms [26]. The appearance of thrombocytopenia and an elevation in the circulation of immature platelets as a consequence of an infection was discovered to increase ICU mortality among septic patients [27]. Each hour of delayed treatment and each extra hour until treatment completion were associated with an extra 4% ICU mortality risk for septic patients [28].

Artificial intelligence has been increasingly used in medical research to achieve better/or early clinical diagnoses and suggest appropriate treatments. Machine learning (ML) is particularly useful for predicting outcomes in intensive care settings because it can integrate complex information from different sources relatively easily [29]. Although ML approaches can process a large number of complex and multidimensional information, the likelihood of implementing an algorithm is reduced with the number of parameters taken into consideration. Essentially, in relation to the external validity of the machine learning tool, it would be useful to limit the number of parameters to only common parameters [24]. Typically, machine learning models can process many data sources, such as biomarkers, ventilation settings, blood pressure, and medications. However, the availability of all these data may differ significantly between ICU units across healthcare systems; for example, a highly equipped ICU can generate a larger volume of data compared to others. Nonetheless, the use of machine learning tools should not be limited to large amounts of data for sepsis patients generated by high-end ICUs only.

ML methods can predict hospital ICU mortality in sepsis patients [21]. It is essential to investigate and analyze the predictability of machine learning algorithms to determine which model is more accurate at forecasting the chance of ICU mortality, hence, can offer an effective treatment for patients. In predicting hospital ICU mortality of patients with sepsis, a machine learning approach appeared to be outperforming conventional clinical decision rules [22]. However, most previous prediction models for mortality require a large number of variables, including the underlying disease, laboratory data, and clinical parameters. This is particularly true for sepsis ICU patients, with a complex disease trajectory which generates a vast array of data that need to be used in clinical decision making. To predict ICU mortality, ICU length of stay, and severity among septic patients, a study employed three machine learning algorithms (Logistic Regression (LR), Random Forest (RF), and eXtreme Gradient Boosting (XGBoost)), where the RF classifier demonstrated the best classification results for prediction of the severity of sepsis (True Positive = 0.65, True Negative = 0.73, F1 measures = 0.72, AUC = 0.79) [27]. Similarly, for mortality predication, RF illustrated better performance (true positive = 0.50, true negative = 0.84, F1 measures = 0.66, AUC = 0.74) along with prediction of ICU Length of stay (sensitivity = 0.50, specificity = 0.84, F1 score = 0.66, AUC = 0.74). Moreover, Synthetic Minority Over-sampling Technique (SMOTE) was conducted for the minority group to make the dataset balance which increased the sensitivity to 0.49 from 0.13 [30].

The purpose of this study is to develop and compare machine learning models using Extra Trees (ET), Random Forest (RF), and Support Vector Classifier (SVC) for predicting mortality among sepsis ICU patients using their ICU patient records. A machine learning prediction model could benefit physicians in their decision-making on better treatment strategies for sepsis patients during their stay in ICUs and to avoid sepsis ICU mortality.

## 2. Methods

### 2.1. Database Description/Study Population

The data were obtained from the eICU Collaborative Research Database of 208 hospitals in the United States with 335 units where patients were hospitalized between 2014 and 2015. The database is populated with information from multiple critical care units across the continental United States. To comply with the safe harbor provision of the US Health Insurance Portability and Accountability Act (HIPAA), all tables have been de-identified. Included in these provisions is the deletion of all protected health information. Additionally, hospital and unit identifiers have been removed to safeguard the confidentiality of contributing organizations. Included in the data are vital signs, laboratory measurements, medications, APACHE components, care plan information, admission diagnosis, patient history, time-stamped diagnoses from a structured problem list, and analogously chosen procedures. This research used 23,479 out of the total of 200,859 instances to obtain the final findings. These samples were selected using a specific inclusion criterion, where we scanned the primary and secondary diagnosis of the patients and only chose the ones who had sepsis listed. Later on, based on each patient’s proportion of missing to charted data, the data points to be used were selected. The diagnosis of medical difficulties in the dataset was mapped using the International Classification of Diseases (ICD) code for the identification of particular diseases based on ontology. Table 1 shows the information for the dataset used in the study. Initially, the sample size was 23.47 K; for the study, 21,806 ICU septic patients and up to 106 variables were included. Men accounted for 51.87% and females for 48.13%. The dataset was imbalanced with regard to ICU mortality (alive and dead). The dataset contains 82.30% (17,948 cases) of ICU septic patients who are alive and 17.70% (3858 cases) of deceased patients. This disparity in the dataset caused the model’s predictions to be skewed toward the majority of instances (e.g., alive) [31]. An appendix (Supplementary Table S1) is now provided, containing a comprehensive list of all 106 variables included in the analysis, their clinical definitions, and measurement units. Throughout the manuscript, tables and figures reference these standardized definitions to enhance readability and interpretability.

Although this study utilized retrospective data from the eICU Collaborative Research Database (2014–2015), which may limit capturing the latest changes in clinical practices, this dataset remains a robust and comprehensive multicenter ICU database. Future studies should consider validating our findings using more recent datasets to ensure continued relevance

### 2.2. Experimental Design

Using preprocessed data, prediction models for predicting ICU mortality were developed using Python software 3.9, and the Scikit-learn package (version 0.20) was used for the supervised machine learning algorithms that were trained, validated, and tested using five-fold cross-validation. We employed a rigorous five-fold cross-validation methodology, training models on 80% of data and testing on the remaining 20%, repeated five times to provide robust internal validation. Nonetheless, future studies should include external validation using independent cohorts to confirm model generalizability.

#### 2.2.1. Data Preprocessing

Initially, the total number of cases within the dataset was 23,479 with 450 variables. A total of 1673 cases were excluded due to missing values over 60%. In order to achieve proper performance and accuracy in the prediction of machine learning models, 101 variables were eliminated either due to having missing values or being duplicated. Thus, the number of cases was reduced to 21,806 as the study aims to focus on ICU mortality among septic patients. Of those 21,806 ICU patients with sepsis, 3858 (17.70%) were deceased, and 17,948 (82.30%) were alive. Table 2 refers to the descriptions of the Top 30 variables.

To ensure robustness and prevent data leakage, all data preprocessing steps—including missing value imputation and normalization—were performed exclusively within the training folds during the five-fold cross-validation. Specifically, after excluding samples with more than 60% missing data, we applied median imputation to the remaining features, computing the median values only from the training subset in each fold. These values were then used to impute missing entries in the corresponding test subset (Supplementary Figure S1).

For normalization, min-max scaling was employed to ensure compatibility across models, especially Support Vector Machines (SVM), which are sensitive to input feature scales. The minimum and maximum values were computed from the training data in each fold and applied to scale both training and test sets consistently, thus avoiding data leakage. This approach preserved the distribution of the unseen test data and ensured a fair evaluation across folds.

An appendix (Supplementary Table S1) is now provided, containing a comprehensive list of all 106 variables included in the analysis, their clinical definitions, and measurement units. Throughout the manuscript, tables and figures reference these standardized definitions to enhance readability and interpretability

#### 2.2.2. Method of Statistical Analysis

For statistical analysis and a thorough examination of the data, we employed the chi-square test. The chi-square measure is computed between the goal and anticipated values, and the features with the greatest chi-square values can be chosen. Intuitively, if a characteristic is independent of the aim, it is ineffective for categorizing observations [32]. The chi-square test is represented by

(1) $$\mathrm{Chi}-\mathrm{Square}\;\mathrm{test},\;X^{2}=\sum_{i=1}^{n}\frac{{{(O}_{i}-E_{i})}^{2}}{E_{i}}$$

#### 2.2.3. Feature Ranking Techniques

A minimal number of features does not adequately reflect the data, whereas a high number of features might cause the model to over-fit. Therefore, it is essential to determine the significance of a characteristic prior to training. Using three-feature ranking algorithms, the remaining 105 markers of the 106 columns for our study were ranked.

Feature ranking was conducted using three ensemble-based methods: XGBoost, Random Forest, and Extra Trees. Importantly, to avoid data leakage, feature importance ranking and selection were performed independently within each training fold of the cross-validation process. This means that for each fold, features were ranked based only on the training subset, and the selected top N features were then applied to train the model and tested on the corresponding held-out test subset. This strategy ensures that no information from the test fold influenced feature selection or model training, thereby preserving the integrity of the evaluation process.

The feature ranking process was repeated across all folds, and aggregated rankings were used to determine the most frequently selected features. These high-ranking features were then reported in the result.

Extreme Gradient Boosting (XGBoost) is a well-known machine learning algorithm that employs a gradient-boosting framework. While XGBoost is predominantly utilized for predictive modeling tasks, it also includes a built-in method for identifying the most significant features in a dataset.

Random forest is an algorithm for machine learning that incorporates the predictive and classifying power of multiple decision trees. In random forest, feature selection refers to the process of identifying a subset of features that are most pertinent or informative for the prediction task at hand. This measure of importance is typically computed during the random forest’s construction.

Extra Trees (Extremely Randomized Trees) is an approach to feature selection that employs a collection of decision trees to determine the most significant features in a dataset. Each decision tree is developed using a random subset of features and random divisions. It is an extension of the Random Forest algorithm. Extra Trees takes a step further and incorporates additional randomness by selecting split points at random rather than seeking for the optimal split.

#### 2.2.4. Experiments

The dataset exhibited significant class imbalance, with 82.3% of patients labeled as “alive” and 17.7% as “deceased”. To address this, three sampling strategies were employed: under-sampling, over-sampling using SMOTE, and a hybrid approach combining under-sampling with SMOTE-based augmentation.

Crucially, all sampling techniques were applied exclusively to the training data within each fold, while the test data retained its original class distribution. This prevented any distortion of the model’s evaluation and ensured that test performance accurately reflects deployment in a real-world population.

All models trained under these scenarios were evaluated only on untouched, imbalanced test subsets, to preserve real-world representativeness. The techniques are described in detail below.

##### Under-Sampling Method

Using resampling techniques to balance the dataset was one of the most prevalent approaches. The majority class (alive) was randomly reduced in size to match the minority class (deceased). The dataset may be under-sampled or over-sampled when resampling techniques are carried out. Under-sampling is the process of decreasing the number of instances or samples of the predominant target population [33]. For under-sampling, the number of living cases was randomly lowered from 17,948 to 3858 to match the number of deceased cases.

##### Over Sampling Method

It is feasible to conduct over-sampling by increasing the number of minority class instances or samples through the production of new instances or the repetition of some instances. The minority class was synthetically expanded using the SMOTE algorithm. SMOTE is a prime example of over-sampling methods [33].

SMOTE is a method of over-sampling in which the minority class is over-sampled through the creation of “synthetic” examples as opposed to replacement. It generates synthetic examples in a manner that is less application-specific by operating in “feature space” as opposed to “data space.” The minority class is over-sampled by introducing synthetic examples along the line segments connecting any/all of the k nearest neighbors of the minority class [34]. The minority class over-sampled from 3858 to 17,948 to balance the dataset.

##### Under-Sampled Augmented Method

The majority classes were split into subsets, each matched with SMOTE-augmented minority data to maintain balance across training batches. The dataset is disproportional from 82.30% to 17.70%. As a result of the under-sampling procedure, 64.60% of the entire dataset is discarded, resulting in a significant loss of information. However, for the over-sampling procedure, sampling elements from the minority set can cause the model to over-fit to a specific set of data, resulting in inaccurate predictions of future data. To address these two issues, we subdivide the majority class into three subclasses and then over-sample the minority class to achieve parity with each of the three majority subdivisions. For the under-sampled combined with augmented strategy, each subset had 5983 to 3858 instances. The subsets were then balanced using the over-sampling method SMOTE.

#### 2.2.5. Machine Learning Model Development

This study assesses different approaches to machine learning, including MLP Classifier, Linear Discriminant Analysis, XGB Classifier, Random Forest Classifier, Logistic Regression, SVC, Extra Trees Classifier, ADABoost Classifier, KNN Classifier, and Gradient Boosting Classifier. The best three performing algorithms are namely Extra Trees, Random Forest, and SVC. In the next section, a general overview of the best models is presented.

##### Extra Trees (ET)

The Extra Trees (Extremely Randomized Trees) algorithm is an ensemble learning technique that constructs a multitude of decision trees at training time and outputs the average prediction of the individual trees to improve predictive accuracy and control over-fitting. It belongs to the family of ensemble methods which base their predictions on a combination of several base estimators in order to improve robustness over a single estimator. The Extra Trees algorithm diverges from other tree-based ensemble methods, such as Random Forest, in the way it splits nodes. It randomizes both the attributes and cut points chosen for node splitting, thereby adding an additional layer of randomness compared to Random Forest. This approach reduces the variance of the model, as it averages multiple highly varied individual models. Moreover, due to this randomization, Extra Trees can be more efficient to train compared to models that require more fine-tuned splitting criteria [35].

##### Random Forest (RF)

**Figure 1:**
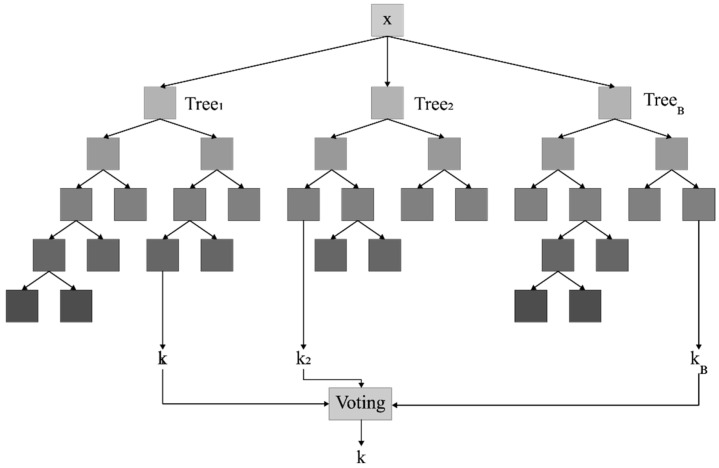
A general architecture of random forest.

Random Forest is one of the machine learning algorithms that can be used for classification and regression, among other functions. In clinical problems, training the Random Forest model with historical electronic health records enables it to predict patient outcomes. A Random Forest is comprised of a large number of randomly sampled, small decision trees known as estimators; each estimator generates its own predictions, and in the training process, numerous decision trees are constructed using the bootstrapping technique. Given that there are numerous trees in the forest, each of which make a decision, the Random Forest relies on a voting mechanism; consequently, the average of the votes is calculated [36]. Figure 1 depicts the Random Forest model’s architecture [36,37].

##### Support Vector Classifier (SVC)

Support Vector Machines (SVMs) are a class of supervised learning models that are widely used for classification and regression tasks in machine learning. When specifically talking about classification, the model is often referred to as Support Vector Classifier (SVC). SVC operates by finding the hyperplane that best separates different classes in the feature space. The most optimal hyperplane is the one that maximizes the margin between the classes, where the margin is defined as the distance between the hyperplane and the nearest points from each class, known as support vectors [38].

The mathematical foundation of SVCs involves solving a convex optimization problem to find the hyperplane that maximizes the margin. This is typically achieved using techniques like quadratic programming. SVC can be extended to nonlinear classification problems using the kernel trick, a method that involves mapping input features into a high-dimensional space where a linear separator is possible [39].

Extra Trees (ET) and Random Forest (RF) classifiers were configured with 100 decision trees each, and a maximum depth parameter set to ‘None’ to allow nodes to expand until all leaves were pure or contained fewer than two samples. For the Support Vector Machine (SVM) classifier, a radial basis function (RBF) kernel was used. Optimization of the SVM was achieved using grid-search with cross-validation for hyperparameter tuning (C and gamma parameters).

### 2.3. Performance Metrics

We employed the receiver operating characteristic (ROC) curves and area under the curve (AUC) along with Precision, Sensitivity, Specificity, Accuracy, and F1-Score to assess the performance of the classifiers. In addition, we used five-fold cross-validation, which culminates in a split of 80% and 20% for the train and test sets, respectively, and according to the fold number, this procedure is repeated five times to evaluate the complete dataset.

We used per-class weighted metrics and overall precision since the number of instances varied across classes. Additionally, we employed the AUC value as an additional assessment statistic. Five assessment measures (weighted sensitivity or recall, specificity, precision, total accuracy, and F1-score) are represented mathematically in Equations (4)–(8):

(2) $$Accuracy_{class\_i}=\frac{TP_{class\_i}+TN_{class\_i}}{TP_{class\_i}+TN_{class\_i}+FP_{class\_i}+FN_{class\_i}}$$

(3) $$Precision_{class\_i}=\frac{TP_{class\_i}}{TP_{class\_i}+FP_{class\_i}}$$

(4) $$\frac{Recall}{Sensitivity_{class_{i}}}=\frac{TP_{class_{i}}}{TP_{class_{i}}+FN_{class_{i}}}$$

(5) $$F1\_score_{class_{i}}=2\frac{Precision_{class_{i}}\times Sensitivity_{class_{i}}}{Precision_{class_{i}}+Sensitivity_{class_{i}}}$$

(6) $$Specificity_{class\_i}=\frac{TN_{class\_i}}{TN_{class\_i}+FP_{class\_i}}$$

where *class__i_* = Alive and Dead Classes

Here, true positive, true negative, false positive, and false negative are represented as TP, TN, FP, and FN, respectively.

The performance of different models was assessed using different evaluation metrics with 95% confidence intervals (CIs). Accordingly, the CI for each evaluation metric was computed as follows:

$$r=z\sqrt{metric(1-metric)/N}$$

where *N* is the number of test samples and *z* is the level of significance that is 1.96 for 95% CI.

## 3. Results

### 3.1. Statistical Analysis

The descriptive analysis is shown in Table 3 and Table 4 for continuous and discrete variables, respectively.

The table indicated that our data are higher among males compared to females being 51.8% and 48.2% respectively. The data are well balanced for age groups of 50–64, 65–79, and >80. There are five major ethnicities listed and the rest taken into a sub-category. The data have around 24.6% patients with intubation and 29.6% for vent. Around 9.3% of them were on dialysis.

### 3.2. Feature Ranking

In the initial investigation, XGBoost outperformed the other two approaches. Hence, XGBoost was utilized to rank features since the outcomes were superior when employing this feature ranking. The primary role of its weight is to rank the independent variables, which are then organized into a decision tree to forecast the result. If the variable weights were improperly recognized, they would be reinserted into the second decision tree to produce an accurate model. This model may also be applied for regression and classification [40,41].

**Figure 2:**
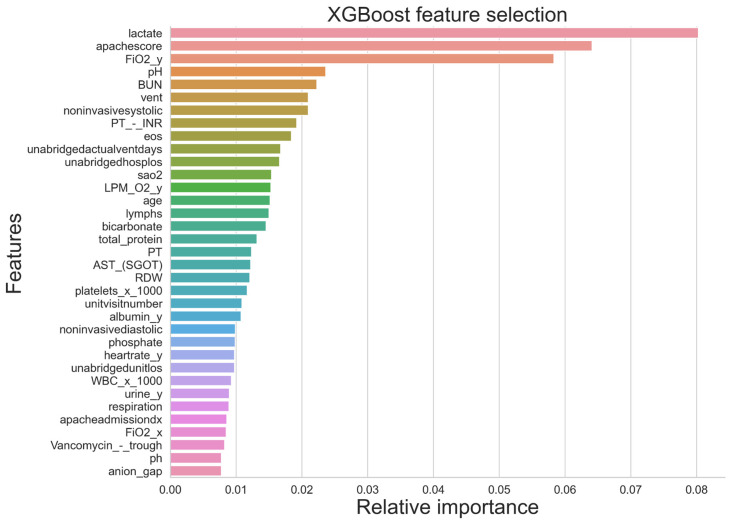
Feature ranked according to XGBoost feature selection algorithms.

Figure 2 displays the Top 35 characteristics that have been assessed by XGBoost. Albumin and Gender were identified by XGBoost as the most significant clinical and demographic characteristics. Table 3 and Table 4 present the feature analysis results using the XGBoost method. Variables marked with *p* < 0.05 indicate statistically significant predictive power for mortality outcomes, thus emphasizing their clinical importance in the prognosis of sepsis mortality. These statistically significant features should receive closer clinical attention due to their stronger predictive relevance.

### 3.3. Experiments

Using a set of predictor variables, algorithms were utilized to predict the ICU mortality of a patient with sepsis. Using three ML models, the likelihood that a patient would have a high risk of death was predicted. The threshold value was 0.5. In the next section, a summary of the findings from three models utilizing three sampling approaches is provided.

#### 3.3.1. Under-Sampled Method

**Figure 3:**
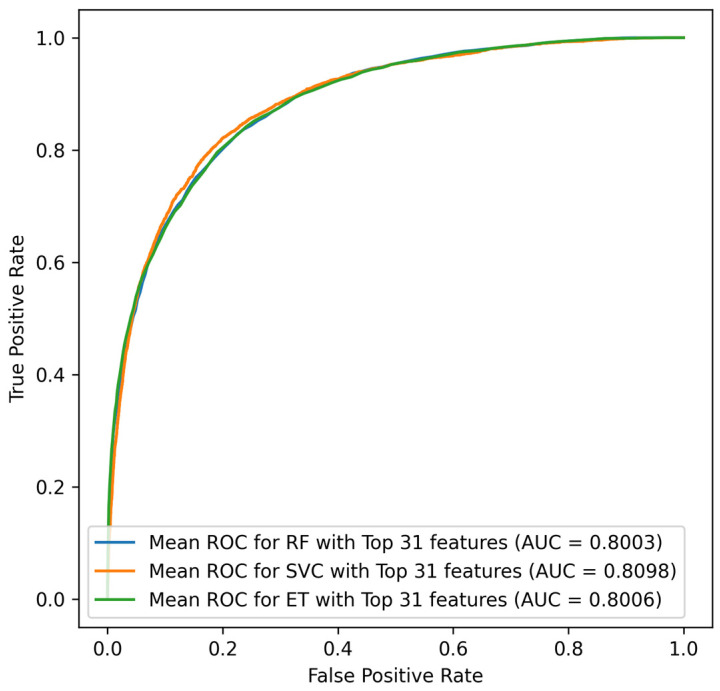
ROC curves for under-sampled techniques for the ET, SVC, and RF classifier.

In the under-sampling procedure, the living cases were randomly under-sampled to 3858 instances, creating a balance between the living and death data. SVC demonstrated superior performance with 80.98% AUC in comparison to the ET and RF classifiers. In the SVC classifier, the confusion matrix for the top 31 features demonstrated the best performance in down-sampling, compared to MLP and RF. The ROC curves in Figure 3 demonstrate the performance of this strategy.

**Figure 4:**
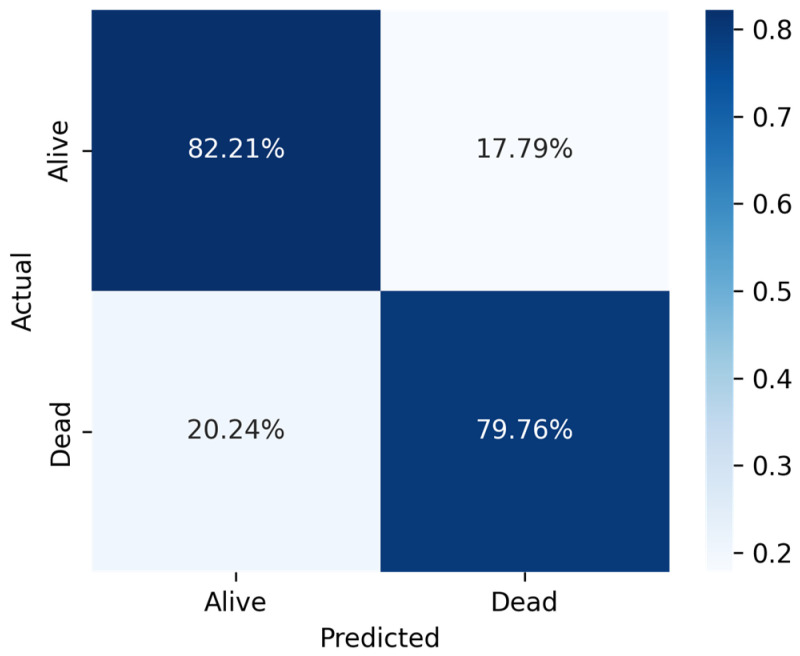
Confusion matrix for SVC classifier for top 31 features on under sampled dataset.

For the under-sampled criteria, the system properly identified 3077 patients as True Negative and incorrectly identified 781 patients as False Positive. In addition, the algorithm incorrectly identified 3193 patients as False Negatives and properly classified 14,755 patients as True Positives. The under-sampled criterion achieved a better AUC score at 80.98% among the three trained models. Table 5 represented the performance scores of the under-sampling technique (Figure 4).

#### 3.3.2. Over-Sampled Method

**Figure 5:**
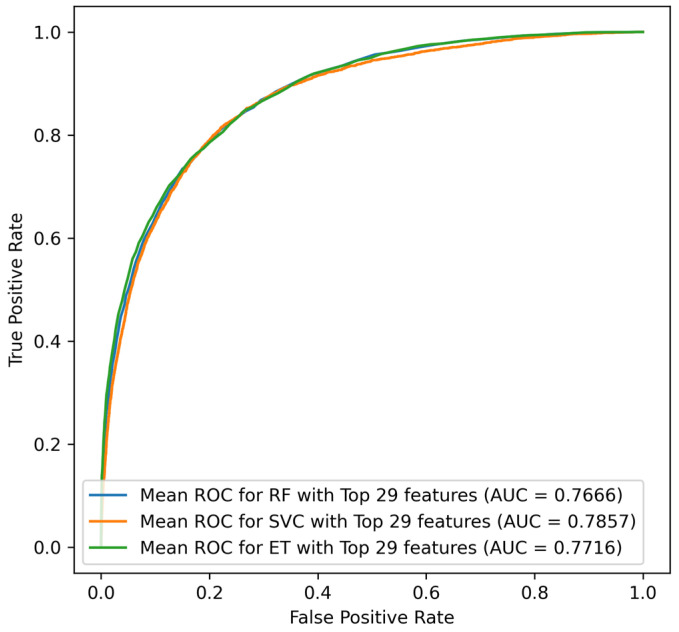
ROC curves for over-sampled techniques for ET, SVC, and RF classifier.

The number of deceased patients was inflated to a total of 17,948 in order to balance the dataset with the number of living cases. SVC classifiers generated superior performance for this approach compared to ET and RF classifiers; for SVC, the combination of the top 29 characteristics produced superior performance with an accuracy of 82.99%. Figure 5’s ROC curves, which are focused on the most significant attributes, depict the performance of the over-sampling strategy.

**Figure 6:**
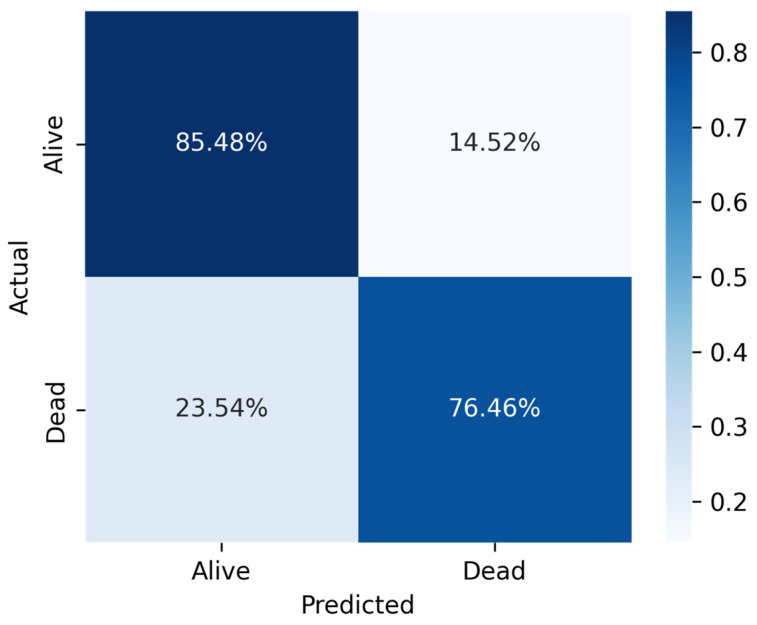
Confusion matrix for SVC classifier for top 29 features on over-sampled dataset.

The over-sampling method’s confusion matrix for the SVC model with the top 24 features indicates that the algorithm accurately identified 2767 instances as TN and incorrectly classified 1091 cases as FP, whereas only 2618 cases were labeled as FN and 15,330 cases as TP. The AUC score for the over-sampled technique was 78.56% for the same models. Table 6 provides performance scores for the confusion matrix as shown in Figure 6.

#### 3.3.3. Under-Sampled Combined with Augmented Method

**Figure 7:**
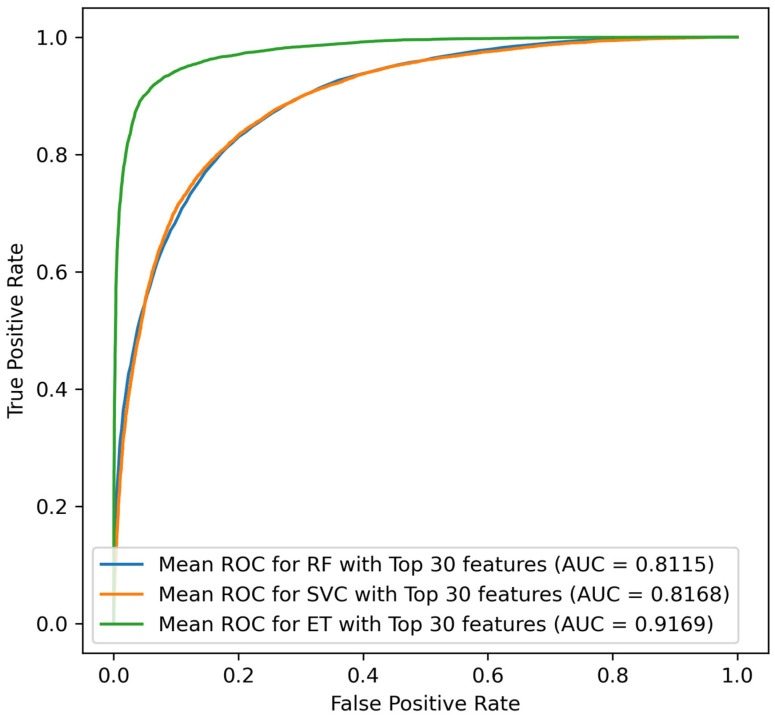
ROC curves for combined sample techniques for the ET, SVC, and RF classifier.

From the prior investigations, it was concluded that SVC was generating the best results for this dataset. For this strategy, the top 30 features on ET and an average subset accuracy of 91% produced the best result. Figure 7 depicts the ROC curve for the leading characteristic. The superior performance of the Extra Trees classifier with combined sampling techniques (under-sampling and SMOTE augmentation) may be attributed to its inherent ensemble structure that enhances robustness against over-fitting, and its ability to better manage imbalanced datasets by leveraging synthetic data generation methods effectively. Unlike RF, ET incorporates more randomized splits, reducing variance. Compared to the SVM classifier, ET handles large feature spaces and noisy datasets more efficiently, contributing significantly to its improved predictive performance.

The average scores across the subsets are presented in Table 7.

**Figure 8:**
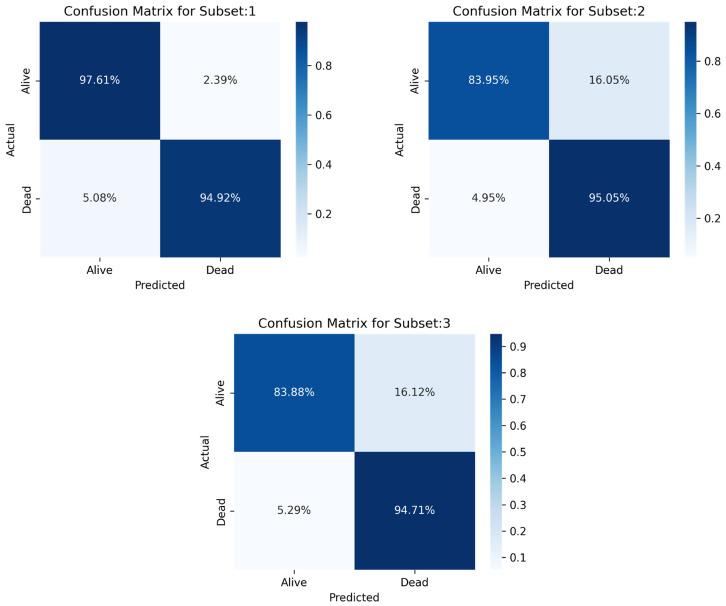
Confusion matrix for stacking classifier for each subset.

For the combined sampling method, the TN ranged from 94.71% to 95.05% between the three subsets, whereas the TP ranged from 83.88% to 97.61%. The confusion matrix generated from each dataset is illustrated in Figure 8.

Table 8 Compares the overall accuracies and weighted average performance of the subsets of the combined method.

Compared with previous studies utilizing similar machine learning methods for sepsis mortality prediction, our ET classifier with combined sampling achieved an accuracy of 0.85 (AUC: 0.89), surpassing or equaling recently reported outcomes. For instance, previous studies by Gao et al. (2024) [42] achieved an accuracy of 0.90 (AUC: 0.89) using RF, and Zhang et al. (2024) [43] reported the accuracy of 0.89 (AUC: 0.88) using gradient-boosting approaches and Kim et al. (2024) [44] reported the accuracy of 0.64 using TabNet. Our approach highlights the advantages of integrating sampling techniques effectively to improve model performance further.

## 4. Discussion

Sepsis costs the healthcare system more money when its occurrence rates rise. Through a multidisciplinary relationship between public health specialists, healthcare providers, patients, and the community, sepsis prevention problems are addressed. Thus, it is clear how crucial it is to use machine learning techniques for accurate and early mortality prediction.

According to the research, male septic patients had a greater mortality rate than female septic patients (*p* value = 0.0480). Additionally, the study findings supported the existing evidence base in that sepsis mortality was more common in men than in women (*p* value < 0.001); however, this may have been a result of the genders’ unadjusted baseline rates of death, which were 54.2% for male and 45.8% for female.

Glomerular filtration rate (GFR), despite information about dialysis and urine output being provided, was one factor that might have been linked to the prediction of ICU mortality for sepsis patients but was not included in the dataset. In addition, such information was required to estimate the stage of kidney disease as it is important in determining organ failure and severity of sepsis.

Sepsis mortality is influenced by a variety of factors, including differences in genetic make-up and biological characteristics, which are strongly influenced by race. Numerous studies have shown a strong correlation between Black people’s death and their race; one of these studies showed that Black people had a case fatality rate of 32%, which was much higher than that of Hispanic and White people (30.4% and 29.3%, respectively) [45]. These results, however, did not support the conclusions of the current investigation because Hispanic ethnicity was associated with a higher mortality risk than Black ethnicity (OR: 1.118, 95% CI: 0.765–2.074 *p* value 0.660); however, this association was found to be statistically insignificant. Another study with contradicting findings found that mortality among White people was higher than that of Black people (OR: 0.85; 95% CI: 0.84–0.86) [46].

Patients from different ethnic groups were included in the US dataset that was used in the study. Although some patients from the Middle East may have been included in the other ethnicity groups designated as “other/unknown”, the model can still be trusted even if Middle Easterners were not among the ethnic groups studied because it was built using training data from 80% of the data cases using the available predictors for the outcome. The other 20% of data cases, which were not part of the training set, were test data, suggesting that external validity was taken into account.

In order to anticipate future events with a high degree of accuracy and to demonstrate the incremental validity, machine learning (ML) models of prediction produce parameters based on the relationship of various factors in the training set. Additionally, it enables the evaluation of clinical factors to determine whether or not they influence mortality prediction. It is tailored as a technique to reduce mortality among ICU septic patients because prediction models are typically constructed to tailor the intensity of characteristics into the preventive. Additionally, it aids in resource allocation so that ICU mortality in the future can be managed.

In this study, ML models were created to forecast ICU mortality for patients with sepsis using a variety of indicators. The primary objective of the study was to assess the efficacy of various machine learning algorithms, of which the three best models are deployed (MLP, RF, and LR) to predict outcomes using patient electronic health information. In the last study, a stacking classifier with three boosting classifiers acting as primary models and LR serving as the final estimator was used.

In comparison to MLP and RF, LR recorded a good prediction with low biases and less deference between accuracy and precision performance. The key findings of this study demonstrated that among the three machine learning algorithms, LR showed the highest performance measures across the three sampling techniques (Sensitivity = 0.808070, Specificity = 0.970375, Precision = 0.918418, Accuracy = 0.917757, F1-score = 0.916139, AUC = 0.88).

This study has several advantages. The fact that the data were collected at random gave the study one of its many advantages, reducing the likelihood of biases and allowing generalization of the results. Additionally, three machine learning algorithms were used, by employing three different sampling strategies to allow the data to be over- and under-sampled. This approach was simple to deploy, economical, quick, and non-invasive because it did not require direct interaction with the patients. The relationship between clinical variables was examined with the help of a general physician to make sure that the variables and test results made clinical sense in order to prevent any misleading or wrong results.

Although our model demonstrates reliable mortality predictions using single-entry ICU data, incorporating longitudinal data could significantly enhance model performance. Predictions at different time points (initial admission, post-resuscitation, and chronic stages of critical illness) can offer tailored insights, thus guiding timely clinical interventions. Future studies are encouraged to examine the temporal dimensions of ICU mortality prediction systematically.

### Limitation

This study demonstrated that just one machine learning method had an accuracy level of 91.77% for predicting ICU mortality. Therefore, the goal of predicting ICU mortality cannot be discounted because it helps healthcare professionals identify patients at high risk. Predicting ICU mortality will be advantageous not only on an individual basis but also on a social, departmental, and corporate level, which can speed up the process of making a strategic decision. Furthermore, by understanding effective resource allocation strategies, healthcare officials can utilize machine learning as a guide to establish new programs based on the proportion of high-risk persons in a specific region or for each health organization.

The technique of data gathering was one of the major weaknesses of the study. It was a secondary database which was publicly accessible for credential account holders. There might be ambiguities in relation to data collection, data process, and data storage that needed to be reviewed and deleted. All of the clinical and demographic data were obtained by registered nurses working in the ICU. Another drawback was the absence of information on the use of pacemakers. As a result, we were unable to rule out patients whose temperatures were below 22 °C, which can be a sign of cardiac arrest; nevertheless, since they were still alive, it was presumed that a pacemaker had been used [47]. Similar to this, some patients who had a heart rate below 45 bpm, which is often indicative of Third-Degree Atrioventricular Block and were still alive, were not disqualified from receiving a pacemaker [48,49].

Algorithm bias is one of the limitations that could be found in such studies, despite the fact that artificial intelligence (AI) is known to lessen the impact of human biases. Algorithm bias is the unfair outcome that a computer system can create due to some repeatable and systematic errors, such as the privilege of one group over the other (living cases over the deceased). In other words, it could happen if the study’s algorithm yields biased results as a result of false assumptions made during the machine learning process [50,51]. However, it was determined that because three sampling criteria were used, the likelihood of this kind of bias is minimal.

## 5. Conclusions

This study shows that clinical predictors using machine learning algorithms can predict ICU mortality. This study found that the proposed stacking model with the Logistic Regression algorithm can achieve Sensitivity of 80.81%, Specificity of 97.04%, Precision of 91.84%, Accuracy of 91.78, F1-score of 91.61%, and AUC of 88% in predicting ICU mortality. The stacking algorithm outperforms individual classifiers in terms of forecasting sepsis ICU mortality. In terms of applying prediction models to improve healthcare decision-making, the study findings would be helpful to guide better management of sepsis ICU patients to reduce ICU mortality. Future studies could conduct other study designs or model analysis techniques to overcome the limitations of this study.

## Figures and Tables

**Table 1:** Hospital level information.

Hospital Level Factor	Number of Hospitals	Number of Patients (%)
Bed capacity		
<100	46 (22.12%)	12,593 (6.27%)
100–249	62 (29.81%)	41,966 (20.89)
259–499	35 (16.83%)	45,716 (22.76%)
≥500	23 (11.06%)	75,305 (37.49%)
Unknown	42 (20.19)	25,279 (12.59%)
Teaching status		
False	189 (90.87%)	149,181 (74.27%)
True	19 (9.13)	51,678 (25.73%)
Region		
Midwest	70 (33.65%)	65,950 (32.83%)
Northeast	13 (6.25%)	14,429 (7.18%)
South	56 (26.92%)	60,294 (30.02%)
West	43 (20.67%)	46,348 (23.07%)
Unknown	26 (12.50%)	13,838 (6.89%)

**Table 2:** Description of variables.

Variable Name	Description
Categorical	
1. apacheadmissiondx	283 categories
2. hospitaldischargestatus	Alive: 17,948
	Expired: 3858
Continuous	
1. lactate	Min = 0.0, Max = 32.2
2. apachescore	Min = −1.0, Max = 211.0
3. FiO2_y	Min = 0.0, Max = 550.75
4. pH	Min = 0.0, Max = 8.0
5. BUN	Min = 1.75, Max = 263.0
6. vent	Min = 0.0, Max = 1.0
7. noninvasivesystolic	Min = 45.0, Max = 199.57
8. PT-INR	Min = 0.79, Max = 16.56
9. eos	Min = 0.0, Max = 46.65
10. unabridgedactualventdays	Min = 1.0, Max = 347.0
11. unabridgedhosplos	Min = 0.1632, Max = 345.81
12. sao2	Min = 7.0, Max = 100.0
13. LPM_O2_y	Min = 0.0, Max = 840.0
14. lymphs	Min = 0.0, Max = 99.28
15. bicarbonate	Min = 4.0, Max = 52.0
16. total_protein	Min = 1.35, Max = 12.7
17. PT	Min = 8.8, Max = 160.5
18. AST_(SGOT)	Min = 4.2, Max = 26,127.0
19. RDW	Min = 4.975, Max = 37.08
20. platelets_x_1000	Min = 2.0, Max = 1143.33
21. albumin_y	Min = 0.6, Max = 6.3
22. noninvasivediastolic	Min = 20.39, Max = 133.08
23. phosphate	Min = 0.3, Max = 169.0
24. heartrate_y	Min = 24.4, Max = 172.67
25. unabridgedunitlos	Min = 0.1666, Max = 345.79
26. WBC_x_1000	Min = 0.038, Max = 414.32
27. urine_y	Min = −1360.0, Max = 529,518.0
28. respiration	Min = 0.0, Max = 106.0

**Table 3:** Baseline characteristic of septic patients (continuous variables).

Features	Mean	SD	Maximum	Minimum	Z-Scores	*p* Values
Lactate	2.59	2.10	16.21	0.30	5.41	<0.05
Apache score	85.04	28.28	173.00	−1.00	4.36	<0.05
FiO2	47.29	12.93	100.00	20.95	5.90	<0.05
pH	7.35	0.08	7.57	6.93	3.51	<0.05
BUN	34.82	20.47	125.29	4.31	4.41	<0.05
Vent	0.72	0.45	1.00	0.00	1.47	0.14
Noninvasive systolic	119.43	15.76	175.94	68.76	5.74	<0.05
PT-INR	1.50	0.67	7.05	0.90	6.27	<0.05
eos	1.39	1.42	11.33	0.00	5.24	<0.05
Unabridged vent days	7.35	16.17	347.00	1.00	1.27	0.20
Unabridged hosp. LOS	17.27	21.26	345.81	0.85	5.54	<0.05
sao2	96.47	2.41	99.90	64.99	4.39	<0.05
LPMO2	13.01	48.55	840.00	0.00	4.07	<0.05
Age	63.56	15.01	90.00	20.00	2.09	0.04
Lymphs	11.07	7.73	69.00	0.73	5.30	<0.05
Bicarbonate	24.45	4.59	40.40	7.81	6.19	<0.05
Total protein	5.88	0.93	10.70	3.35	2.43	0.01
PT	16.61	6.32	56.27	9.30	5.51	<0.05
AST_(SGOT)	183.88	834.47	15,804.00	6.50	5.12	<0.05
RDW	16.41	2.39	26.63	12.59	3.31	<0.05
Platelets	210.32	107.83	674.44	11.25	4.68	<0.05
Unit visit number	1.12	0.46	5.00	1.00	0.11	0.91
Albumin	2.67	0.59	4.80	0.98	3.60	<0.05
Noninvasive diastolic	64.33	9.93	105.73	28.21	5.36	<0.05
Phosphate	3.50	1.27	12.18	1.00	2.80	<0.05
Heart rate	90.55	13.78	144.83	53.83	4.83	<0.05
Unabridged unit LOS	10.35	19.84	345.79	0.24	2.04	0.04
WBC	13.21	5.84	58.23	0.45	3.22	<0.05
Urine	17,564.86	31,025.50	529,518.00	0.00	5.44	<0.05
Respiration	21.08	4.18	40.39	7.60	2.97	<0.05
Apache admissiondx	61.53	24.76	84.00	0	1.37	0.17

**Table 4:** Baseline characteristic of septic patients (categorical and binary variables).

Variable Name	Frequency	Percentage
1. Age categorical	(0) 18–34: 1174	5.4%
	(1) 35–49: 2039	9.3%
	(2) 50–64: 5851	26.8%
	(3) 65–79: 7069	32.4%
	(4) ≥80: 4413	20.2%
2. Gender	F: 10,493	48.2%
	M: 11,311	51.8%
3. Ethnicity	African American: 2125	9.7%
	Asian: 453	2%
	Caucasian: 16,704	76.6%
	Hispanic(H): 1076	4.9%
	Native America (NA): 194	0.9%
	Other/unknown (O/Unk): 1244	5.9%
4. Intubated	0: 16,485	75.6%
	1: 1603	24.6%
5. Vent	0: 14,223	65.2%
	1: 1926	29.6%
6. Dialysis	0: 19,363	88.8%
	1: 606	9.3%
7. Meds	0: 20,131	92.3%
	1: 93	1.4%
8. Ptcawithin24h	0: 18,038	99.8%
	1: 10	0.2%

**Table 5:** Performance measures of under sampling.

Under-Sampling						
	Accuracy	Precision	Sensitivity	Specificity	F1-Score	AUC
SVC	81.78% ± 0.51%	49.08% ± 0.66%	79.76% ± 0.53%	82.21% ± 0.51%	60.76% ± 0.65%	80.98% ± 0.52%
ET	81.33% ± 0.52%	48.29% ± 0.66%	78.10% ± 0.54%	82.02% ± 0.51%	59.68% ± 0.65%	80.06% ± 0.53%
RF	81.05% ± 0.52	47.84% ± 0.66%	78.43% ± 0.55%	81.61% ± 0.51	59.43% ± 0.65%	80.03% ± 0.53%

**Table 6:** Performance measures of over-sampling.

Over-Sampling						
	Accuracy	Precision	Sensitivity	Specificity	F1-Score	AUC
SVC	82.99% ± 0.49%	51.38% ± 0.66%	71.72% ± 0.59%	85.41% ± 0.47%	59.87% ± 0.65%	78.56% ± 0.54%
ET	86.25% ± 0.46%	60.73% ± 0.65%	63.09% ± 0.64%	91.23% ± 0.38%	61.89% ± 0.64%	77.16% ± 0.58%
RF	85.59% ± 0.47%	58.68% ± 0.65%	62.83% ± 0.64%	90.49% ± 0.39%	60.69% ± 0.65%	76.66% ± 0.56%

**Table 7:** Performance analysis for the combined sample technique.

	Precision	Recall	F1-Score
Class 0	96.41% ± 0.25%	88.48% ± 0.42%	92.28% ± 0.35%
Class 1	84.16% ± 0.48%	94.89% ± 0.29%	89.20% ± 0.41%
Accuracy	90.99% ± 0.38%		
AUC	91.69% ± 0.37%		

**Table 8:** Performance measures of for each subset for the combined sampling criteria.

	Accuracy	Precision	Sensitivity	Specificity	F1-Score	AUC
Subset 1	96.56% ± 0.24%	96.24% ± 0.25%	94.92% ± 0.29%	97.61% ± 0.20%	96.56% ± 0.24%	96.26% ± 0.25%
Subset 2	88.30% ± 0.43%	79.25% ± 0.54%	95.04% ± 0.29%	83.95% ± 0.49%	88.30% ± 0.43%	89.50% ± 0.41%
Subset 3	88.13% ± 0.43%	79.12% ± 0.54%	94.71% ± 0.29%	83.89% ± 0.49%	88.13% ± 0.43%	89.30% ± 0.41%

## Data Availability

The dataset utilized for this study is made publicly available by Pollard et al. [31].

## Supplementary Materials

The following supporting information can be downloaded at: https://www.mdpi.com/article/10.3390/jcm14103495/s1, Figure S1: Workflow diagram to illustrate data processing and training & validation of ML models; Table S1: Definition of Variables.
