# Supplementary material for: Predicting ICU Mortality Among Septic Patients Using Machine Learning Technique
Source: J Clin Med. 2025 May 16;14(10):3495. doi: 10.3390/jcm14103495 (PMC12111920; doi:10.3390/jcm14103495)
Supplement: Supplementary file 1 [file jcm-14-03495-s001.zip › jcm-3559009-Supplementary Materials.pdf]

Supplementary Material

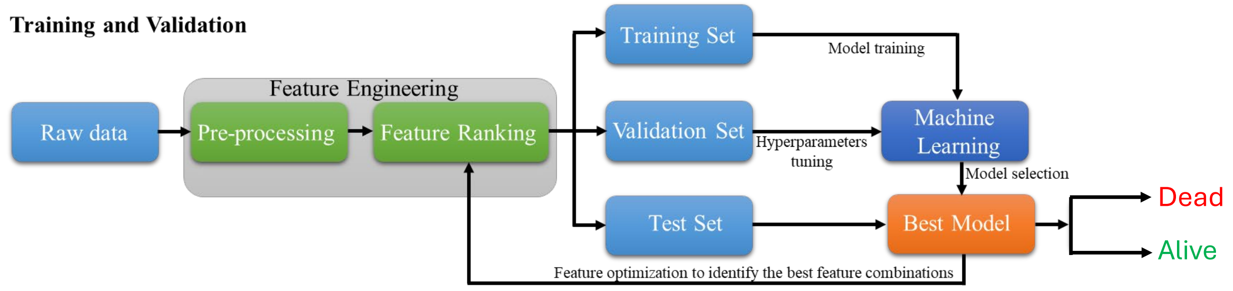

Figure S1: Workflow diagram to illustrate data processing and training & validation of ML models.

Supplementary Materials

Table S1. Definition of Variables.

**Variable Name**	**Definition**
patientUnitStayID	A unique identifier for a single patient admission to an Intensive Care Unit (ICU).
gender	The recorded gender of the patient (e.g., Male, Female, Other, Unknown).
age	The age of the patient in full years at the time of admission (patients over 89 are often recorded as "> 89").
ethnicity	The recorded ethnicity of the patient (e.g., Caucasian, African American, Hispanic, Asian, Native American, Other/Unknown).
apacheAdmissionDx	The primary reason or diagnosis for the patient's ICU admission.
admissionHeight	The patient's height recorded upon admission, typically in centimeters (cm).
hospitalDischargeStatus	The patient's status upon discharge from the hospital (e.g., Alive, Expired).

unitType	The type of ICU the patient was admitted to (e.g., MICU, SICU, CCU, Neuro ICU).
unitVisitNumber	A number indicating which ICU stay this is for the patient during a single hospitalization (e.g., 1st, 2nd).
unitDischargeStatus	The patient's status upon discharge from the specific ICU (e.g., Alive, Expired).
weight	The patient's weight, typically recorded at admission, in kilograms (kg).
allergy_counts	A derived count representing the number of known allergies for the patient.
vent	An indicator (often 0 or 1) signifying if the patient was mechanically ventilated.
intubated	An indicator (often 0 or 1) signifying if the patient was intubated.
dialysis	An indicator (often 0 or 1) signifying if the patient was receiving dialysis.
eyes	The score for the 'eye opening' component of the Glasgow Coma Scale (GCS), ranging from 1 to 4.
motor	The score for the 'motor response' component of the Glasgow Coma Scale (GCS), ranging from 1 to 6.
verbal	The score for the 'verbal response' component of the Glasgow Coma Scale (GCS), ranging from 1 to 5.
meds	An indicator signifying if GCS scoring was potentially confounded by medication administration.
urine_x	The patient's summed urine output over a 24-hour period, often used in severity scoring.
wbc	The patient's White Blood Cell count, often the value associated with the greatest deviation from normal within the first day.

temperature_x	The patient's body temperature (Celsius), often the value associated with the greatest deviation from normal within the first day.
respiratoryRate_x	The patient's respiratory rate (breaths per minute), often the value associated with the greatest deviation from normal within the first day.
sodium_x	The patient's serum sodium level (mEq/L), often the value associated with the greatest deviation from normal within the first day.
heartRate_x	The patient's heart rate (beats per minute), often the value associated with the greatest deviation from normal within the first day.
meanBp	The patient's mean arterial blood pressure (mmHg), often the value associated with the greatest deviation from normal within the first day.
ph	The patient's arterial blood pH value, often from the Arterial Blood Gas (ABG) result associated with the greatest derangement.
hematocrit	The patient's hematocrit level (%), often the value associated with the greatest deviation from normal within the first day.
creatinine_x	The patient's serum creatinine level (mg/dL), often the value associated with the greatest deviation from normal within the first day.
albumin_x	The patient's serum albumin level (g/dL), often the value associated with the greatest deviation from normal within the first day.
pao2	The partial pressure of oxygen in the patient's arterial blood (mmHg), often from the ABG result associated with the greatest derangement.
pco2	The partial pressure of carbon dioxide in the patient's arterial blood (mmHg), often from the ABG result associated with the greatest derangement.
bun	The patient's Blood Urea Nitrogen level (mg/dL), often the value associated with the greatest deviation from normal within the first day.

glucose_x	The patient's blood glucose level (mg/dL), often the value associated with the greatest deviation from normal within the first day.
bilirubin	The patient's serum bilirubin level (mg/dL), often the value associated with the greatest deviation from normal within the first day.
fio2	The fraction of inspired oxygen being delivered to the patient (%), often from the ABG result associated with the greatest derangement.
physicianSpeciality	The medical specialty of the physician associated with the patient's care (e.g., cardiology, pulmonology).
physicianInterventionCategory	A category indicating the level of intervention authority granted to remote monitoring clinicians.
acutePhysiologyScore	The Acute Physiology Score component calculated as part of a severity of illness scoring system (e.g., APACHE).
apacheScore	The overall calculated score from the APACHE (Acute Physiology Age Chronic Health Evaluation) severity of illness system.
preopMI	An indicator if the patient had a history of Myocardial Infarction before a surgical procedure or within 6 months.
ptcawithin24h	An indicator (0 or 1) if the patient underwent a Percutaneous Transluminal Coronary Angioplasty within the previous 24 hours.
unabridgedUnitLOS	The patient's actual total Length of Stay in the ICU, in days.
unabridgedHospLOS	The patient's actual total Length of Stay in the hospital, in days (may be capped at a maximum value like 50).
unabridgededadactualeventdays	Variable name as provided; specific definition not available in the source text.

urine_y	A measurement of the patient's urine output, potentially recorded at various times or intervals.
_basos	Measurement related to basophils (a type of white blood cell), likely count or percentage.
eos	Measurement related to eosinophils (a type of white blood cell), likely count or percentage.
_lymphs	Measurement related to lymphocytes (a type of white blood cell), likely count or percentage.
_monos	Measurement related to monocytes (a type of white blood cell), likely count or percentage.
_polys	Measurement related to polymorphonuclear leukocytes (neutrophils, a type of white blood cell), likely count or percentage.
ALT (SGPT)	Alanine Transaminase (Serum Glutamic Pyruvic Transaminase) level, an indicator of liver function.
AST (SGOT)	Aspartate Aminotransferase (Serum Glutamic Oxaloacetic Transaminase) level, an indicator of liver function.
BUN	Blood Urea Nitrogen level (mg/dL), an indicator of kidney function.
CPK	Creatine Phosphokinase level, an enzyme found in heart, brain, and skeletal muscle.
FiO2_x	Fraction of Inspired Oxygen (%), often associated with the worst Arterial Blood Gas result in a specific period.
HCO3	Bicarbonate level (mEq/L), typically measured in blood gas or chemistry panels, indicating acid-base balance.
Hct	Hematocrit level (%), the proportion of blood volume occupied by red blood cells.

Hgb	Hemoglobin level (g/dL), the protein in red blood cells that carries oxygen.
MCH	Mean Corpuscular Hemoglobin (pg), the average amount of hemoglobin per red blood cell.
MCHC	Mean Corpuscular Hemoglobin Concentration (g/dL), the average concentration of hemoglobin inside red blood cells.
MCV	Mean Corpuscular Volume (fL), the average volume (size) of red blood cells.
MPV	Mean Platelet Volume (fL), the average size of platelets in the blood.
O2_Sat(%)	Oxygen Saturation level (%), typically measured non-invasively via pulse oximetry (SpO2).
PT - INR	Prothrombin Time - International Normalized Ratio, a measure of blood clotting time.
PTT	Partial Thromboplastin Time (seconds), another measure of blood clotting time.
RBC	Red Blood Cell count (e.g., million cells per microliter).
RDW	Red Cell Distribution Width (%), a measure of the variation in red blood cell size.
Vancomycin - trough	The lowest concentration of the antibiotic Vancomycin in the bloodstream, measured before the next dose.
WBC x 1000	White Blood Cell count, typically expressed as cells per microliter (e.g., 11.5 representing 11,500 cells/uL).
albumin_y	Serum albumin level (g/dL), a type of protein in the blood plasma.
alkaline_phos.	Alkaline Phosphatase level, an enzyme found primarily in the liver and bone.

anion_gap	Anion Gap (mEq/L), a calculated value representing the difference between measured cations and anions in serum.
bedside glucose	Blood glucose level (mg/dL) measured at the patient's bedside, typically using a point-of-care device.
bicarbonate	Bicarbonate level (mEq/L), measured in blood, reflecting acid-base status.
calcium	Serum calcium level (mg/dL), an important electrolyte.
chloride	Serum chloride level (mEq/L), an important electrolyte.
creatinine_y	Serum creatinine level (mg/dL), an indicator of kidney function.
glucose_y	Blood glucose level (mg/dL), typically measured in a laboratory chemistry panel.
lactate	Blood lactate level (mmol/L), often used as an indicator of tissue perfusion or metabolic stress.
magnesium	Serum magnesium level (mg/dL), an important electrolyte.
pH	Acidity or alkalinity level measured in a blood sample, typically arterial blood gas (ABG).
paCO2	Partial pressure of carbon dioxide (mmHg) measured in arterial blood, reflecting ventilation status.
paO2	Partial pressure of oxygen (mmHg) measured in arterial blood, reflecting oxygenation status.
phosphate	Serum phosphate (or phosphorus) level (mg/dL), an important electrolyte.
platelet	Platelet count (e.g., thousand cells per microliter), involved in blood clotting.

potassium	Serum potassium level (mEq/L), an important electrolyte.
potassium_x_1000	Serum potassium level (mEq/L); likely redundant with 'potassium', potentially related to specific scaling or source.
sodium_y	Serum sodium level (mEq/L), an important electrolyte.
total_bilirubin	Total bilirubin level (mg/dL) in the blood, related to liver function and red blood cell breakdown.
total_protein	Total protein level (g/dL) in the blood serum.
troponin_I	Troponin I level (ng/mL), a cardiac biomarker used to detect heart muscle injury.
urinary_specific_gravity	Specific gravity of urine, indicating the concentration of solutes in the urine.
FiO2_y	Fraction of Inspired Oxygen (%) being delivered to the patient, potentially recorded from ventilator or charting.
LPM O2_y	Oxygen flow rate in Liters Per Minute (LPM) being delivered to the patient, typically via nasal cannula or mask.
PEEP_y	Positive End-Expiratory Pressure (cmH2O), a ventilator setting used to keep alveoli open.
RR (patient)	Respiratory Rate (breaths per minute) of the patient, recorded from observation or monitoring devices.
noninvasivesystolic	Systolic blood pressure (mmHg) measured using a non-invasive cuff.
noninvasivediastolic	Diastolic blood pressure (mmHg) measured using a non-invasive cuff.
sao2	Oxygen saturation level (%) measured non-invasively via pulse oximetry (SpO2).

heartrate_y	Heart rate (beats per minute) of the patient, recorded from monitoring devices or physical exam.
respiration_y	Respiratory rate (breaths per minute) of the patient, recorded from monitoring devices.
st1	Measurement related to the ST segment on an electrocardiogram (ECG), Lead 1.
st2	Measurement related to the ST segment on an electrocardiogram (ECG), Lead 2.
st3	Measurement related to the ST segment on an electrocardiogram (ECG), Lead 3.
